# Dyslipidemia may impact initial recovery following arthroscopic rotator cuff repair: a retrospective study

**DOI:** 10.1186/s13018-024-04650-x

**Published:** 2024-03-07

**Authors:** Lei Yao, Xiumei Zhao, Lu Mei, Yinghao Li, Long Pang, Chunsen Zhang, Jian Li, Xin Tang

**Affiliations:** 1 Sports Medicine Center, West China Hospital, Sichuan University, Chengdu, 610041 China; 2 Department of Orthopedics and Orthopedic Research Institute, West China Hospital, Sichuan University, Chengdu, 610041 China; 3 Operating Room, West China Hospital, Sichuan University/West China School of Nursing, Sichuan University, Chengdu, 610041 China; 4 West China School of Nursing, Sichuan University/Department of Orthopedics, West China Hospital, Sichuan University, Chengdu, 610041 China

**Keywords:** Rotator cuff, Arthroscopy, Dyslipidemia, Ortholiposis, Functional outcome

## Abstract

**Background:**

The current literature shows that dyslipidemia can lead to a higher incidence of rotator cuff tears (RCTs) and an increased retear rate after repair. We aimed to evaluate the influence of preoperative dyslipidemia on postoperative pain, patient-reported outcomes (PROs), active range of motion (ROM), and structural integrity.

**Methods:**

A cohort of 111 patients who underwent arthroscopic RCT repair between January 2021 and July 2022, and whose complete preoperative serum lipid data were available within one week prior to surgery was retrospectively reviewed. Dyslipidemia was defined as the presence of an increase or decrease in at least one blood lipid profile (triglycerides, total cholesterol, low-density lipoprotein, high-density lipoprotein, or non-high-density lipoprotein). There were 43 patients in the dyslipidemia group and 68 in the ortholiposis group. Patient evaluations, including pain score, PROs, and ROMs, were conducted preoperatively; at 3 and 6 months postoperatively; and at the last follow-up. Structural integrity was assessed by magnetic resonance imaging (MRI) 6 months after surgery if possible, and Sugaya type 4 or 5 was considered a retear. Propensity score matching (PSM) was used to reduce bias.

**Results:**

The RCT size, surgical technique, preoperative pain status, PROs, and active ROM were comparable between patients with dyslipidemia and those with ortholiposis. Three months after surgery, patients in the dyslipidemia group had worse average PROs (Constant score: *P* = 0.001; ASES score: *P* = 0.012; UCLA score: *P* = 0.015), forward flexion (*P* = 0.012), and internal rotation (*P* = 0.001) than patients in the ortholiposis group did. The difference between the two groups persisted after PSM but disappeared at the sixth month after surgery. No significant differences in pain score, PROs, or active ROMs were detected between the dyslipidemia and ortholiposis groups after a mean follow-up of 24 months. Of the 72 patients who underwent MRI, 4 retears (5.6%) were found, and all were in the ortholiposis group. There was no difference in the rate of retears between the two groups (*P* = 0.291) or with (*P* = 0.495) PSM.

**Conclusions:**

In conclusion, we found that perioperative dyslipidemia may impact initial recovery within the first 3 months following arthroscopic rotator cuff repair but may have no effect on pain, PROs, or active ROMs at a mean 2-year follow-up or rotator cuff integrity at 6 months postoperatively.

*Trail registration* Retrospectively registered.

## Introduction

Rotator cuff tears (RCTs) are common conditions in old individuals who can result in persistent pain and functional impairment. A study by Milgrom et al. [1] suggested that the incidence of RCT increases with age in people over 50 years of age, and asymptomatic rotator cuff disease was present in as many as 50% of dominant shoulders in the seventh decade and in 80% of patients over 80 years of age. Performing rotator cuff repair is appropriate for symptomatic rotator cuff tears that do not improve with conservative treatment. In recent years, numerous attempts to improve postoperative outcomes have been extensively studied by surgeons [2–4].

However, satisfactory postoperative outcomes are influenced by numerous factors, both extrinsic and intrinsic [5]. Research on various factors influencing clinical outcomes and tendon integrity after rotator cuff repair has been analyzed in the past and has not recently gained more prominence [5–7]. The landmark article published by Lin et al. [7] confirmed that hyperlipidemia, a kind of dyslipidemia, is an independent risk factor for RCTs. More studies have begun to focus on postoperative outcomes in RCT patients with dyslipidemia. Recently, there has been an increasing focus on more detailed and diverse results in this field. The majority of studies confirm the negative impact of dyslipidemia on clinical outcomes after rotator cuff repair [8–10]. In addition, few studies have focused on the effect of dyslipidemia on RCT postoperative patient-reported outcomes (PROs) [8, 11]. To our knowledge, no studies have determined whether the early postoperative recovery process in patients with dyslipidemia is consistent with that in ortholiposis patients. Recently, enhanced recovery after surgery has been a hot topic. It is very important to determine the factors that affect early postoperative rehabilitation and apply relevant interventions to improve the postoperative quality of life of patients.

Therefore, the objective of this retrospective study was to evaluate the influence of preoperative dyslipidemia on postoperative pain, PROs, active range of motion (ROM), and structural integrity and to determine whether dyslipidemia affects the process of recovery in patients.

## Methods

### Study design

This was a retrospective, hospital-based, cohort study. We compared the clinical and radiological outcomes of patients who underwent arthroscopic RCT repair with or without dyslipidemia between January 2021 and July 2022. All of the surgeries were performed by one senior shoulder surgeon. Ethical approval for this study was approved by our institutional review board (West China Hospital, 2020-934).

### Population selection

The inclusion criteria were as follows: (1) symptomatic full-thickness isolated supraspinatus tear confirmed by magnetic resonance imaging (MRI) and arthroscopic examination; (2) aged between 40 and 80 years; (3) has complete serum lipid results within one week before operation; (4) had a minimum follow-up of 12 months; and (5) had complete medical files, including pre- and postoperative examination results. The exclusion criteria were (1) partial tear or massive tears (>5 cm); (2) revision surgery; (3) tears unrepaired or repaired without suture anchors; (4) concomitant shoulder stiffness, glenohumeral arthritis, glenohumeral instability, calcific tendinitis, local infection, etc.; (5) severe cervical disorders or axillary nerve palsy; or (6) uncontrolled diseases such as cancer or infection.

The patients were subsequently allocated into two groups according to their preoperative serum lipid profiles. The diagnosis and segregation of patients in the ortholiposis group and dyslipidemia group were determined through hematological screening within one week before the operation, established with reference to our local guidelines, and drawn from the Joint Committee on the Chinese Guidelines for Lipid Management [12]. Dyslipidemia was defined as the presence of an increase or decrease in at least one blood lipid profile (total cholesterol ≥ 6.2 mmol/L, triglycerides ≥ 2.3 mmol/L, low-density lipoprotein ≥ 4.1 mmol/L, high-density lipoprotein ≤ 1.0 mmol/L, and non-high-density lipoprotein ≥ 4.9 mmol/L).

### Surgical technique

All operations were performed by one senior shoulder surgeon. The lateral decubitus position was adopted, and the affected shoulder joint was stretched. After general anesthesia, a standard posterior portal and an anterior central portal were established, and an arthroscopic examination of the shoulder joint was performed. Subacromial decompression was performed for a better surgical field of view. The lateral and anterior lateral portals were subsequently established, and extensive bursectomy and release of tendon adhesions were performed. During the examination, acromial morphology and the long head of the biceps tendon were assessed. The surgeon chooses to perform acromioplasty, tenodesis, or tenotomy. The type of RCT was evaluated and classified based on the method of DeOrio and Cofield [13]. Afterwards, the footprint was debrided with a motorized bur to improve the healing of the tendon-bone interface after repair. The rotator cuff was repaired with suture anchors using a single-row or double-row technique with regard to tear size and shape. After the water-tight, tension-free repair, an intra-articular evaluation of the construct was performed.

### Postoperative rehabilitation

All patients received standardized postoperative rehabilitation. The patient was discharged the day after surgery and visited the surgeon's clinic for rehabilitation guidance every 2–3 weeks until the third month after surgery and again at six months and each year after surgery.

Following surgical intervention, the affected arm was immobilized in an abduction pillow at 30° for a duration of 6–8 weeks according to the tear size. During this time, patients were permitted to perform passive range-of-motion exercises of the hand, elbow, and wrist. At two weeks postoperatively, passive forward flexion, and abduction were initiated with guidance at the surgeon's clinic. The patients were allowed to perform internal and external rotation exercises after an additional 4 weeks. Upon removal of the pillow, patients progressed to active assisted and active shoulder movements and were able to resume normal daily activities without limitations. It should be noted that no strengthening or resistance exercises were allowed prior to 3 months postoperatively.

### Patient evaluation

The following data were collected preoperatively: age, sex, body mass index (BMI), symptom duration, visual analog scale (VAS) score for pain, PROs (Constant score, American Shoulder and Elbow Surgeons [ASES] score, University of California, Los Angeles [UCLA] score), and active ROM (active forward flexion, external rotation, and internal rotation). Postoperative outcomes were evaluated and recorded during regular outpatient follow-up. The VAS score was recorded at 1, 3, and 6 months postoperatively and at the latest follow-up, and PROs and active ROM were recorded at 3 and 6 months postoperatively and at the latest follow-up. Structural integrity was evaluated by the surgeon and an experienced radiologist using 6-month postoperative MRI if available, and retear was defined as Sugaya classification types 4 and 5 [14].

### Statistical analyses

The data analysis for this study was generated using IBM SPSS Statistics for Windows, version 22.0 (IBM Corp., Armonk, N.Y., USA). A descriptive analysis was performed in which continuous variables were described by the mean and standard deviation and categorical data were defined by percentages. The normality of the variables was tested with Kolmogorov–Smirnov test, and the Levene test was used to test for equal variances. The independent *t* test was employed to test the differences between groups when the continuous variances satisfied a normal distribution. Otherwise, the Mann-Whitney U test was adopted. The chi-square test was performed to determine the differences in categorical variables. To compensate for the difference in patient characteristics between the two groups, we used propensity score matching (PSM), a statistical method, to screen patients in the ortholiposis group matched 1:1 with similar age, sex, BMI, diabetes, and follow-up time. Statistical significance was set at *P* < 0.05.

## Results

### Patient characteristics

**Fig. 1:**
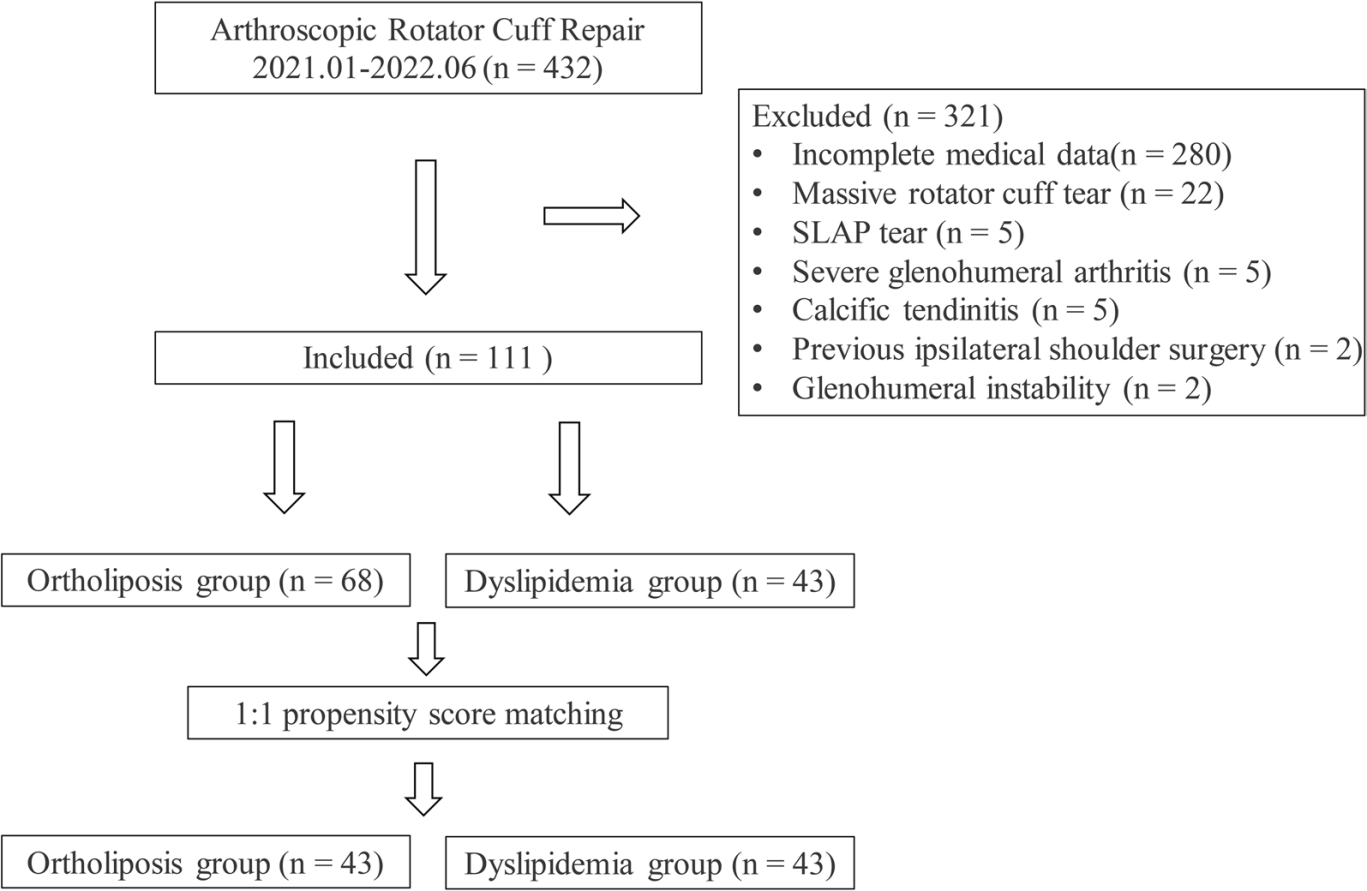
Flowchart of patient inclusion. SLAP, superior labrum anterior and posterior

Figure 1 depicts the flow chart of the 111 patients included in the final analysis and the 86 patients included after PSM according to the established inclusion and exclusion criteria. Patient characteristics and intraoperative findings are shown in Tables 1 and 2, respectively. Patient characteristics, except BMI and diabetes status, were comparable between the two groups. Patients with dyslipidemia had a higher mean BMI (25.7 ± 3.0 vs. 24.5 ± 2.9, *P* = 0.041) and a higher incidence of diabetes (39.5% vs. 22.1%, *P* = 0.048) than did those in the ortholiposis group. No significant differences were found regarding tear findings, including tear size, repair technique, anchor number, biceps tenotomy/tenodesis, or acromioplasty. After PSM, patient and tear characteristics were comparable between the groups, although the number of anchors used in the dyslipidemia group was higher than that in the ortholiposis group.

**Table 1:** Patient characteristics

	Before matching			After matching		
	Ortholiposis n = 68	Dyslipidemia n = 43	*P*	Ortholiposis n = 43	Dyslipidemia n = 43	*P*
Age, yr	58.5 ± 8.4	57.7 ± 11.5	0.686	57.7 ± 8.2	57.7 ± 11.5	0.974
Sex						0.822
Female	42 (61.8)	27 (62.8)	0.914	28 (65.1)	27 (62.8)	
Male	26 (38.2)	16 (37.2)		15 (34.9)	16 (37.2)	
Affected side			0.447			0.348
Left	22 (32.4)	11 (25.6)		15 (34.9)	11 (25.6)	
Right	46 (67.6)	32 (74.4)		28 (65.1)	32 (74.4)	
Body mass index, kg/m^2^	24.5 ± 2.9	25.7 ± 3.0	0.041	24.8 ± 3.1	25.7 ± 3.0	0.189
Symptom duration, mon	21.8 ± 39.6	17.7 ± 28.1	0.550	20.8 ± 45.2	17.7 ± 28.1	0.704
Trauma history	16 (23.5)	13 (30.2)	0.434	11 (25.6)	13 (30.2)	0.631
Diabetes	15 (22.1)	17 (39.5)	0.048	13 (30.2)	17 (39.5)	0.365
Follow-up time, mon	24.7 ± 4.3	23.2 ± 5.0	0.086	25.0 ± 4.7	23.2 ± 5.0	0.091

Data are presented as n (%) or mean ± SD unless otherwise indicated

**Table 2:** Tear characteristics

	Before matching			After matching		
	Ortholiposis n = 68	Dyslipidemia n = 43	*P*	Ortholiposis n = 43	Dyslipidemia n = 43	*P*
Tear size			0.775			0.830
Small	5 (7.4)	2 (4.7)		3 (7.0)	2 (4.7)	
Medium	44 (64.7)	27 (62.8)		28 (65.1)	27 (62.8)	
Large	19 (27.9)	14 (32.6)		12 (27.9)	14 (32.6)	
Repair technique			0.798			0.596
Single-row	14 (20.6)	8 (18.6)		10 (23.3)	8 (18.6)	
Double-row	54 (79.4)	35 (81.4)		33 (76.7)	35 (81.4)	
Anchor no	2.4 ± 1.1	2.7 ± 1.1	0.115	2.2 ± 0.9	2.7 ± 1.1	0.026
Biceps tenotomy/tenodesis	22 (32.4)	14 (32.6)	0.982	14 (32.6)	14 (32.6)	0.999
Acromioplasty	64 (94.1)	43 (100)	0.157	39 (90.7)	43 (100)	0.116

Data are presented as n (%) or mean ± SD unless otherwise indicated

### Outcome scores

**Fig. 2:**
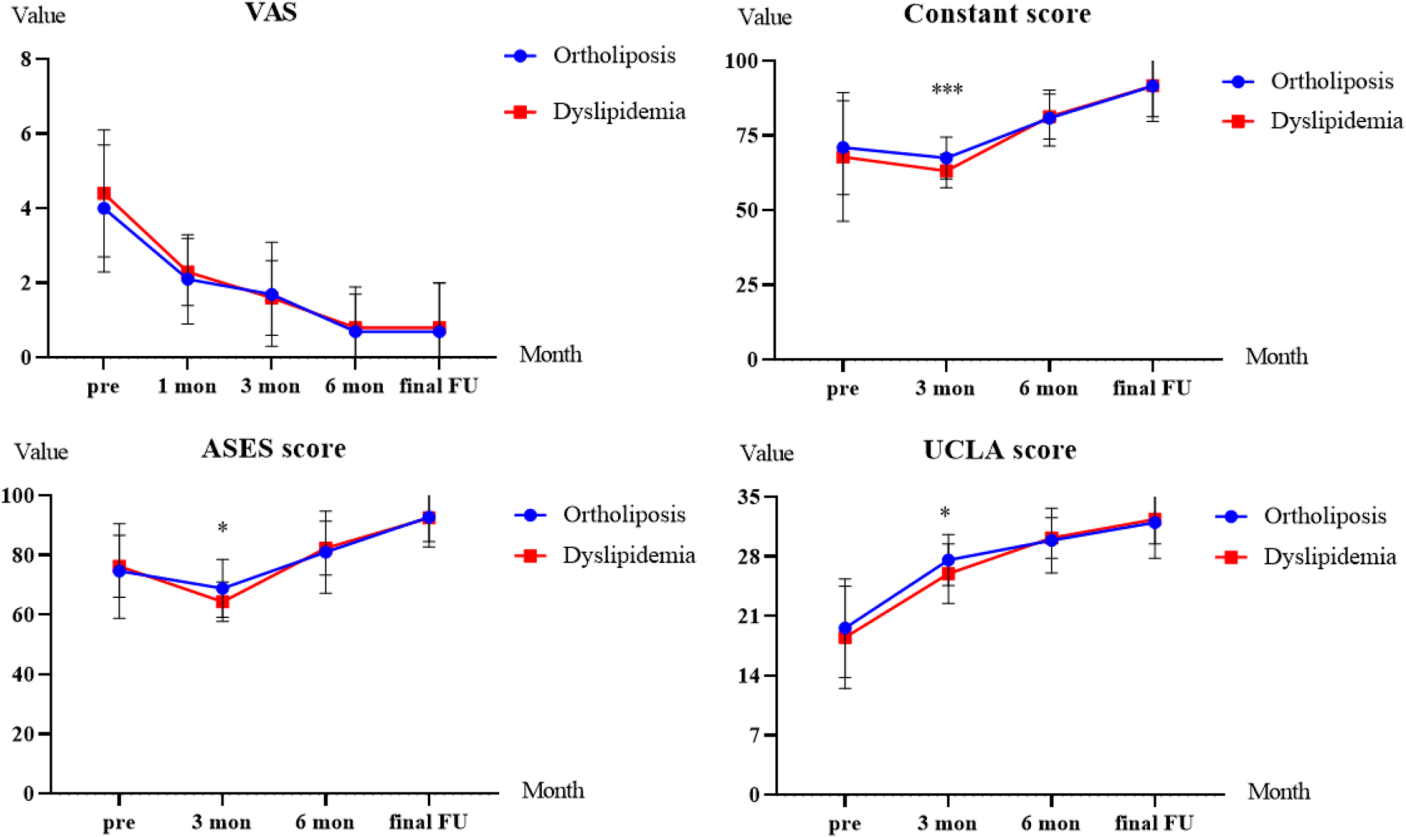
Progression of 4 primary outcome measures in the dyslipidemia vs ortholiposis groups over months. FU, follow-up; VAS, visual analog scale for pain; ASES, American Shoulder and Elbow Surgeons; UCLA, University of California, Los Angeles

**Fig. 3:**
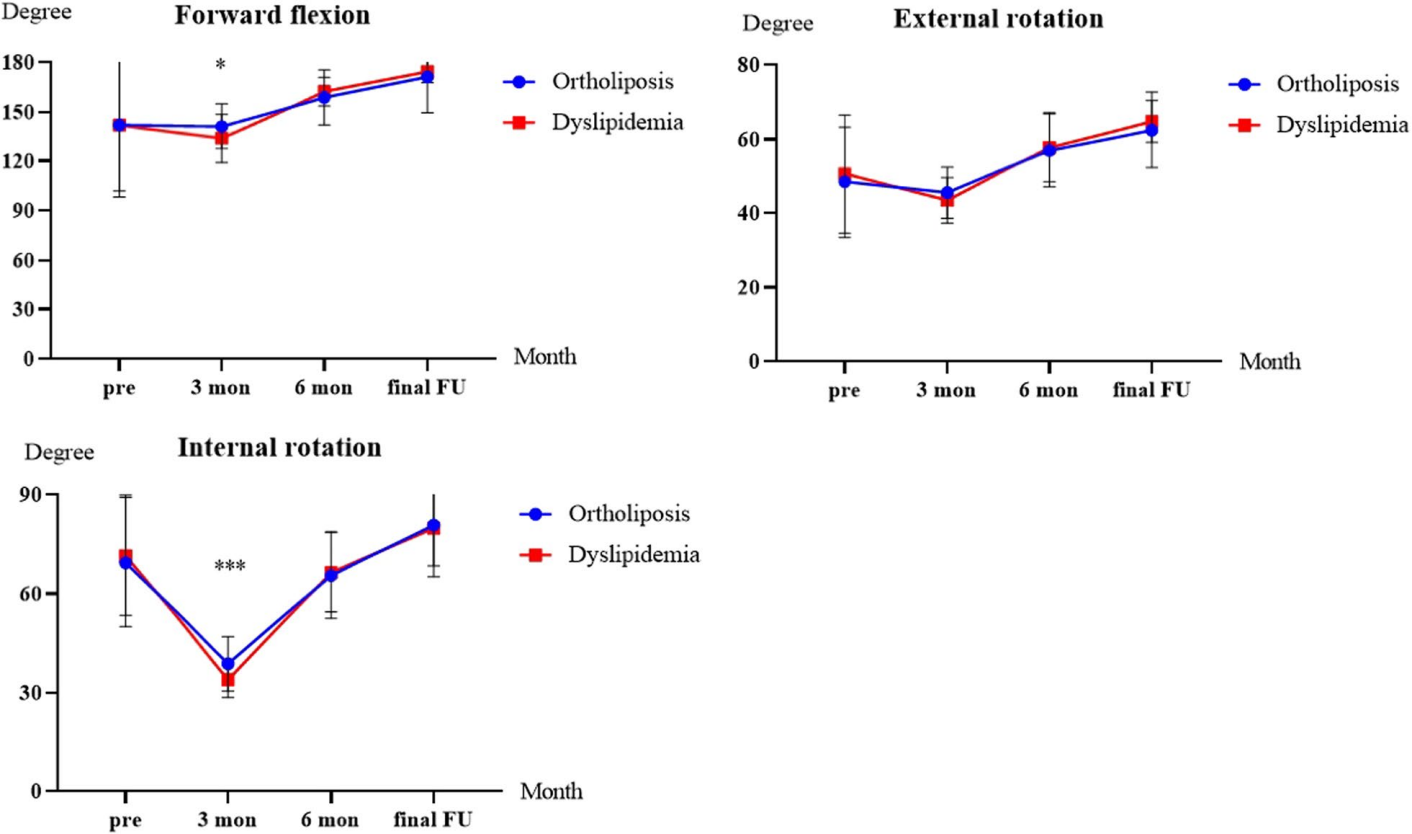
Progression of 3 active range of motion outcome measures in the dyslipidemia versus ortholiposis groups over months. FU, follow-up

The preoperative and postoperative clinical outcomes, including the VAS score for pain, PROs, and active ROM, are shown in Table 3. The series of preoperative and postoperative changes in pain, PROs, and ROMs for all patients are shown in Figs. 2 and 3, respectively.

**Table 3:** Clinical outcomes before propensity score matching

	Ortholiposis group			Dyslipidemia group			*P*
	Preoperative	Postoperative	*P*	Preoperative	Postoperative	*P*	
VAS score for pain	4.0 ± 1.7	0.7 ± 1.3	< 0.001	4.4 ± 1.7	0.8 ± 1.2	< 0.001	0.677
Functional scores							
Constant	71.0 ± 15.7	91.6 ± 11.9	< 0.001	67.9 ± 21.5	91.7 ± 10.4	< 0.001	0.977
ASES	74.7 ± 15.8	92.8 ± 10.0	< 0.001	76.3 ± 10.4	92.5 ± 8.0	< 0.001	0.874
UCLA	19.6 ± 5.8	32.0 ± 4.2	< 0.001	18.5 ± 6.0	32.4 ± 2.9	< 0.001	0.593
Range of motion							
External rotation	51.0 ± 11.3	62.5 ± 10.2	< 0.001	53.1 ± 12.6	64.8 ± 5.6	< 0.001	0.184
Internal rotation	69.5 ± 19.6	80.8 ± 15.6	< 0.001	71.6 ± 18.2	79.9 ± 11.3	< 0.001	0.736
Forward flexion	145.2 ± 34.6	171.2 ± 21.6	< 0.001	146.1 ± 35.5	174.4 ± 6.7	< 0.001	0.342

Data are presented as the mean ± SD unless otherwise indicated. *VAS* visual analog scale, *ASES* American Shoulder and Elbow Surgeons, *UCLA* University of California, Los Angeles

*Pain*. Significant improvement in the VAS score from the preoperative period to the postoperative period was found in both groups (ortholiposis group: 4.0 ± 1.7 vs. 0.7 ± 1.3, *P* < 0.001; dyslipidemia group: 4.4 ± 1.7 vs. 0.8 ± 1.2, *P* < 0.001) (Table 3). No significant differences were found between the 2 groups at any time point before or after PSM (Fig. 2, Tables 3, 4).

**Table 4:** Clinical outcomes after propensity score matching

	Ortholiposis group			Dyslipidemia group			*P*
	Preoperative	Postoperative	*P*	Preoperative	Postoperative	*P*	
VAS score for pain	3.8 ± 1.6	0.7 ± 1.2	< 0.001	4.4 ± 1.7	0.8 ± 1.2	< 0.001	0.578
Functional scores							
Constant	71.2 ± 14.9	90.3 ± 14.4	< 0.001	67.9 ± 21.5	91.7 ± 10.4	< 0.001	0.609
ASES	75.9 ± 13.3	92.4 ± 9.9	< 0.001	76.3 ± 10.4	92.5 ± 8.0	< 0.001	0.968
UCLA	20.1 ± 5.9	31.6 ± 4.9	< 0.001	18.5 ± 6.0	32.4 ± 2.9	< 0.001	0.328
Range of motion							
External rotation	51.5 ± 12.0	61.6 ± 11.7	< 0.001	53.1 ± 12.6	64.8 ± 5.6	< 0.001	0.058
Internal rotation	68.8 ± 19.4	80.2 ± 17.6	< 0.001	71.6 ± 18.2	79.9 ± 11.3	< 0.001	0.913
Forward flexion	142.6 ± 36.1	169.1 ± 26.3	< 0.001	146.1 ± 35.5	174.4 ± 6.7	< 0.001	0.199

Data are presented as the mean ± SD unless otherwise indicated. *VAS* visual analog scale, *ASES* American Shoulder and Elbow Surgeons, *UCLA* University of California, Los Angeles

*PROs.* The preoperative and postoperative Constant score, ASES score, and UCLA score showed no significant differences between the 2 groups, but RCT repair significantly improved the PROs in the two groups (*P* < 0.001) (Tables 3, 4). However, 3 months after the operation, the dyslipidemia group had worse PROs than did the ortholiposis group (Constant score: 63.2 ± 5.7 vs. 67.5 ± 7.0, *P* < 0.001; ASES score: 64.4 ± 6.6 vs. 68.9 ± 9.7, *P* = 0.012; UCLA score: 26.0 ± 3.5 vs. 27.6 ± 3.0, *P* = 0.015). After PSM, the dyslipidemia group showed worse Constant score than did the ortholiposis group (63.2 ± 5.7 vs. 67.6 ± 7.7, *P* = 0.005), while the statistical significance of the differences in UCLA and ASES scores between the groups disappeared. These differences were no longer observed after 6 months.

*Active ROM.* Both groups showed significant improvement in active ROMs at the last follow-up (*P* < 0.001) (Tables 3, 4). There was no difference in external rotation between the two groups at any time point. However, 3 months after surgery, the ortholiposis group of patients performed better in forward flexion and internal rotation (forward flexion: 141.2 ± 13.8 vs. 133.8 ± 14.8, *P* = 0.012; internal rotation: 38.7 ± 8.2 vs. 33.9 ± 5.4, *P* < 0.001). These differences disappeared at subsequent follow-up. After PSM, the dyslipidemia group showed worse active ROMs than did the ortholiposis group (external rotation: 46.6 ± 6.3 vs. 43.4 ± 6.1, *P* = 0.029; internal rotation: 38.6 ± 9.1 vs. 33.9 ± 5.4, *P* = 0.007), while the statistical significance of forward flexion between groups disappeared (139.4 ± 12.3 vs. 133.8 ± 14.8, *P* = 0.077).

*Structural integrity.* MRI was performed for 72 patients (64.9%) in the study cohort: 47 (69.1%) in the ortholiposis group and 25 (58.1%) in the dyslipidemia group. There was no difference in the proportion of patients who underwent in postoperative MRI between the two groups (*P* = 0.238), and the baseline data of the patients who underwent postoperative MRI were comparable. A total of 4 patients (5.6%) were found to have RCT retears, 3 of whom had large tears and 1 of whom had a medium tear before surgery. Interestingly, they were all from the ortholiposis group, but there was no significant difference in the retear rate compared with that in the dyslipidemia group (8.5% vs. 0, *P* = 0.291). After PSM, no difference was found between the groups (6.7% vs. 0%, *P* = 0.495).

## Discussion

A retrospective cohort study was conducted to examine the effect of preoperative dyslipidemia on functional and structural outcomes following arthroscopic RCT repair. The main finding of this study is that perioperative dyslipidemia may impact initial recovery for the first 3 months following arthroscopic rotator cuff repair but may have no effect on pain, PROs, or active ROMs at a mean 2-year follow-up or rotator cuff integrity at 6 months postoperatively. To our knowledge, this is the first study to examine dyslipidemia in patients with early recovery patterns after RCT repair.

Dyslipidemia has been extensively studied as a risk factor for preoperative RCT [7, 15]. The odds of RCT developing in patients with dyslipidemia has been reported to be at least 2 times greater than that in patients without dyslipidemia [7]. In addition to a higher risk of disease, dyslipidemia also appears to affect the outcome of RCT repair. Research by Zeng et al. [11] found that RCT patients with dyslipidemia had worse preoperative PROs; however, statin-treated dyslipidemia patients did not have poorer 2-year PROs compared with those in the control group after RCT surgery. This study did not find an effect of dyslipidemia on preoperative PROs or ROM, nor did it find a difference in PROs or active ROM between the two groups at the last follow-up. In our study, patients with dyslipidemia had a higher proportion of diabetes and a larger BMI, which is consistent with the epidemiological characteristics of patients with dyslipidemia, further enhancing the generalizability of this study. We also performed PSM to reduce the bias associated with BMI and diabetes incidence. However, an interesting finding that may be worth the attention of surgeons is that patients with dyslipidemia seem to experience a slower recovery process, and they generally have lower PROs and worse active ROMs in the first three months after surgery with or without PSM. In addition, Amit et al. [8] prospectively found that the retear rate and postoperative fatty infiltration were comparable between the hyperlipidemia and control groups. Zeng et al. [11] also proposed that good dyslipidemia control through conscientious use of statins should be advocated preoperatively to retain favorable outcomes after arthroscopic RCT surgery. Taken together, these results suggest that patients may benefit from well-controlled serum lipid levels, which is similar to the finding of previous study of diabetes [16]. However, additional clinical practice is needed to determine this difference.

Postoperative rotator cuff retear is the most important factor affecting patient satisfaction. Park et al. [10] reviewed 502 patients who underwent RCT repair and concluded that preoperative hypo-high-density lipoproteinemia has a significant association with retear after surgery in large- to massive-sized RCTs. Similar results were found by Garcia et al. [17], who found that hyperlipidemia increased the risk of retearing after RCT, with an odds ratio of 6.5. Cancienne et al. [9] found that moderate and high perioperative total cholesterol and low-density lipoprotein levels were associated with the rate of revision after RCT repair, and statin use appeared to mitigate the need for revision surgery. However, this study found no difference in retear rate between the two groups. Previous study has found that BMI has no direct effect on the characteristics of rotator cuff tenocytes [18]. Another study has suggested that differences in rotator cuff histology may explain differences in retear rates among different patient groups, while some characteristics did not affect rotator cuff histology [19]. This may explain that we did not find a difference in retear rate between the two groups because the rotator cuff histology may be comparable between the two groups. This suggests that it is necessary to further explore the effect of dyslipidemia on rotator cuff by conducting histological tests in patients with dyslipidemia in future studies. The proportion of patients with retears in this study was lower than that previously reported [8–11, 17], and all retears occurred in the ortholiposis group. One possible explanation for these findings could be the relatively rapid recovery of the ortholiposis group. Previous studies have found that more aggressive reentry activity after surgery may lead to higher rates of retear [20–22], which is consistent with our finding that the ortholiposis group had better ROMs and higher PROs at 3 months. In addition, our imaging follow-up time was 6 months postoperatively, which may have led to missed retears because of the shorter follow-up period [2, 23]. Patients (39/111) who refused MRI examination due to satisfactory functional outcomes may have increased the overall retear rates, however, previous studies have demonstrated that PROs were not associated with the integrity of the repaired rotator cuff [5]. Finally, there is also a potential reason that, based on our observations, the patient population in this study had a lower need for higher intensity activities, which may have further reduced the risk of retearing [6].

Studies have found that recovery after RCT involves not only function exercise at a single joint but also whole-body homeostasis [7, 24, 25]. Therefore, personalized treatment plans should also pay attention to the patients with coexisting systemic diseases. Previous studies have demonstrated that patients with dyslipidemia treated with statins can achieve clinical outcomes comparable to those of patients in the ortholiposis group [8, 9, 11]. However, some studies suggest that the type and dose of statins have no effect on the quality of tendon healing [11]. The side effects of statins on the musculoskeletal system are also of concern [26, 27]. Therefore, additional prospective studies are needed to focus on the effect of drug therapy for dyslipidemia on surgical outcomes.

## Limitation

This study is not without limitations. First, selection bias was present in this study due to its retrospective design, and not all patients were included during the study period. We used PSM to reduce bias, and the characteristics and disease-related findings of the two groups of patients were comparable. Subsequent prospective studies could help us better address this issue. Second, this was a relatively short follow-up. A previous study has found that a 1-year follow-up of PROs is sufficient for evaluating the effectiveness after RCT repair [28], and longer follow-up is needed to investigate the effect of dyslipidemia on rotator cuff healing. Third, MRI at 6 months postoperatively was available for 64.9% of the patients in the study cohort. Even if we examined the baseline data of patients who underwent MRI, the results were comparable between groups, which could introduce the possibility of type II errors. A type II error can occur when a study is underpowered to detect a difference between groups. Larger sample sizes and higher MRI review rates should be encouraged in future studies. Finally, information on perioperative oral medications, especially statins, which can affect serum lipid levels, was not recorded. However, as we discussed earlier, well-controlled serum lipid levels may be beneficial for recovery after RCT. Therefore, monitoring serum lipid levels may be more direct than monitoring medication use in clinical practice.

## Conclusions

In conclusion, we found that perioperative dyslipidemia may impact initial recovery within the first 3 months following arthroscopic rotator cuff repair but may have no effect on pain, PROs, or active ROMs at a mean 2-year follow-up or rotator cuff integrity at 6 months postoperatively. Patients may benefit from good perioperative control of their serum lipid levels in RCTs, and additional well-designed prospective experiments could be useful for validating our findings.

## Data Availability

The study data will be available upon request to the corresponding author.

## Abbreviations

RCT: Rotator cuff tear
PRO: Patient-reported outcome
ROM: Range of motion
ASES: American Shoulder and Elbow Surgeons
UCLA: University of California, Los Angeles
VAS: Visual analog scale
MRI: Magnetic resonance imaging
PSM: Propensity score matching
SLAP: Superior labrum anterior and posterior
